# Blood-Count-Derived Inflammatory Markers as Predictors of Response to Biologics and Small-Molecule Inhibitors in Psoriasis: A Multicenter Study

**DOI:** 10.3390/jcm13143992

**Published:** 2024-07-09

**Authors:** Silviu-Horia Morariu, Ovidiu Simion Cotoi, Oana Mirela Tiucă, Adrian Baican, Laura Gheucă-Solovăstru, Hana Decean, Ilarie Brihan, Katalin Silaghi, Viorica Biro, Diana Șerban-Pescar, Ioana Măgureanu, Mircea Ambros, Roxana Ioana Ilcuș, Lavinia Prodan, Andreea Beatrix Bălan, Mădălina Husariu, Dumitrita Lenuta Gugulus, Radu Alexandru Stan, Vlad Voiculescu, Alin Codruț Nicolescu

**Affiliations:** 1 Dermatology Department, George Emil Palade University of Medicine, Pharmacy, Science, and Technology of Targu Mures, 540142 Targu Mures, Romania; 2 Pathophysiology Department, George Emil Palade University of Medicine, Pharmacy, Science, and Technology of Targu Mures, 540142 Targu Mures, Romania; 3 Dermatology Department, Iuliu Hatieganu University of Medicine and Pharmacy, 400012 Cluj-Napoca, Romania; 4 Dermatology-Venereology Discipline, Department of Medical Sciences III, Faculty of Medicine “Grigore T. Popa”, University of Medicine and Pharmacy, 700115 Iași, Romania; 5 Physiology Department, Iuliu Hatieganu University of Medicine and Pharmacy, 400012 Cluj-Napoca, Romania; 6 Department of Dermatology, Dermatology Clinic, Faculty of Medicine and Pharmacy, University of Oradea, 410087 Oradea, Romania; 7 Dermatology Unit, Bistrița Emergency Clinical County Hospital, 420016 Bistrița, Romania; 8 Dermatology Unit, Odorheiu Secuiesc Town Hospital, 535600 Odorheiu Secuiesc, Romania; 9 Dermatology Unit, Eugen Nicoară Reghin Town Hospital, 545300 Reghin, Romania; 10 Dermatology Clinic, Mures Clinical County Hospital, 540342 Targu Mures, Romania; 11 Dermatology Department, Carol Davila University of Medicine and Pharmacy, 050474 Bucharest, Romania; 12 Agrippa Ionescu Emergency Clinical Hospital, 011773 Bucharest, Romania

**Keywords:** biologics, psoriasis, noninvasive, inflammation, blood count

## Abstract

**Background**: Psoriasis is an immune-mediated chronic disorder associated with various comorbidities. Even though biologics and small-molecule inhibitors are the mainstay treatment for moderate-to-severe psoriasis, there is no current consensus regarding which agent should be used for a specific type of patient. This paper aims to test the reliability of blood-count-derived inflammatory markers in assessing treatment response to biologics and small-molecule inhibitors in psoriasis. **Material and Methods**: Bio-naïve adult patients diagnosed with chronic plaque psoriasis fulfilling the inclusion criteria were enrolled. They were divided into study subgroups based on treatment of choice, and blood-count-derived inflammatory markers were analyzed at baseline, three-month, six-month, and at twelve-month visits. **Results**: A total of 240 patients were included. The highest number of patients underwent treatment with ixekizumab. The neutrophil-to-lymphocyte ratio (NLR), platelet-to-lymphocyte ratio (PLR), platelet-to-monocyte ratio (PMR), monocyte-to-lymphocyte ratio (MLR), derived neutrophil-to-lymphocyte ratio (d-NLR), systemic inflammation response index (SIRI), systemic immune inflammation index (SII), and aggregate index of systemic inflammation (AISI) all varied significantly (*p* < 0.005) between the four visits. The psoriasis area severity index (PASI) score correlated with PLR, d-NLR, and SII, while the psoriasis scalp severity index (PSSI) score correlated with AISI and SIRI. More than half of patients reached the target goal of PASI90 at the six-month visit. A total of 77 patients were super-responders, with the highest number undergoing treatment with ixekizumab. Higher baseline values of d-NLR and SIRI are independent predictors of the super-responder status. **Conclusions**: Blood-count-derived inflammatory markers can serve as indicators of treatment response to biologics in psoriasis, while d-NLR and SIRI were independent predictors of super-responders in our study.

## 1. Introduction

Psoriasis is an immune-mediated chronic disorder that significantly impacts patients’ quality of life and is frequently associated with various comorbidities. The most frequent clinical type, chronic plaque psoriasis, is linked to cardiovascular disorders, such as hypertension, myocardial infarction, or atherosclerosis, metabolic syndrome, diabetes mellitus, and depression [1]. Moreover, patients with psoriasis seem to have an increased risk of presenting with associated nonalcoholic fatty liver disease (NAFLD) [2], most likely due to the associated metabolic syndrome and obesity. Also, the hepato-dermal axis was recently described and proposed as being involved in the pathogenesis of this comorbidity [3]. 

Psoriasis etiopathogenesis involves a complex interplay of genetic, environmental, and immune factors. A continuous interaction between dendritic cells, various subsets of T cells, and keratinocytes leads to an increased inflammatory state in psoriasis, defined by high levels of interleukin (IL)-12, IL-23, IL-17, and tumor necrosis factors (TNF)α [4].

Taking into account lesions’ localization and extension, various treatment options are available for psoriasis. Biologics and small-molecule inhibitors targeting altered immune pathways are currently the gold standard for treating moderate to severe psoriasis. When choosing the optimal treatment, patients’ clinical and biological profiles, including comorbidities, should also be taken into account. A comprehensive, multidimensional approach to patients diagnosed with psoriasis is needed, focusing on skin lesions and all aspects of this disease. A proper choice of treatment would prove useful in addressing not only cutaneous lesions but also associated comorbidities. However, there is no current consensus regarding which agent should be used for a specific type of patient. 

In recent years, various blood-count-derived markers have emerged as reliable and easily obtainable markers for systemic inflammation. Apart from being useful in appreciating the evolution and outcome of chronic obstructive pulmonary disorder (COPD) [4], various cancers [5,6,7] and cardiac disorders [8,9] they were also proven to be indicators of activity disease in dermatological disorders. Their utility has been reported so far in bullous disorders [10], hidradenitis suppurativa [11], and atopic dermatitis [12,13]. On the other hand, conflicting data are reported for acne [14,15]. 

Current evidence of psoriasis focuses mainly on two hematological parameters: the neutrophil-to-lymphocyte ratio (NLR) and the platelet-to-lymphocyte ratio (PLR). Other parameters, such as the monocyte-to-lymphocyte ratio or the derived neutrophil-to-lymphocyte ratio (d-NLR) [16], have also been investigated. Even fewer papers have analyzed composite markers, such as the systemic immune index (SII), systemic immune response index (SIRI), and aggregate index of systemic inflammation (AISI) [16,17]. 

Blood-count-derived inflammatory biomarkers have not been extensively studied regarding biological treatment in psoriasis. Data referring to composite markers in this matter are scarce [18], and, to the best of our knowledge, the reliability of AISI and d-NLR has not been assessed so far regarding treatment response to biologics. 

## 2. Materials and Methods

### 2.1. Study Population

This is a multicentric retrospective study performed on adult patients diagnosed with chronic plaque psoriasis in accordance with the Declaration of Helsinki and approved by the Ethics Committee of Mures Clinical County Hospital (no. 3770/05.04.2023). Bio-naïve patients diagnosed with chronic plaque psoriasis originating from Dermatology Departments of various hospitals (Mures Clinical County Hospital; Cluj Napoca Emergency Clinical County Hospital; Agrippa Ionescu Emergency Clinical Hospital of Bucharest; Sfantul Spiridon Emergency Clinical County Hospital of Iasi, Elias Emergency University Clinical Hospital Bucharest; Emergency Clinical County Hospital of Bistrita Nasaud; Town Hospitals of Odorheiu Secuiesc and Reghin) were enrolled in this study. 

Bio-naïve patients undergoing any biological or small-molecule inhibitors treatment were included in this study if complete, appropriate data were available at treatment initiation, at the three-month, six-month, and twelve-month follow-up. Data regarding demographics, comorbidities, disease activity, and laboratory parameters were collected for enrolled patients. Disease activity was assessed using the Psoriasis Area Severity Index (PASI) score, and when special sites were involved, the Nail Psoriasis Area Severity Index (NAPSI) and the Psoriasis Scalp Severity Index (PSSI) were used. Concomitant psoriatic arthritis was also checked for. Pediatric patients, those with incomplete data, and those benefiting from a therapeutical switch at a certain point were excluded from the analysis. Patients’ data were collected using the hospitals’ electronic databases.

### 2.2. Blood Analysis and Biomarkers

Blood samples were obtained by venipuncture from the upper extremities, more specifically the median cubital vein; when it was not reachable, the cephalic or basilic veins were used. Whole blood venous samples were collected in the morning after an overnight fast for all patients, following each hospital’s drawing blood protocol. Next, the obtained samples were analyzed by spectrophotometry or flow cytometry using an automatic hematology analyzer. Laboratory investigations were performed as per the requirements of our National Psoriasis Protocol, which states that bloodwork should be carried out in psoriatic patients receiving biologics as follows: before the initial visit and three months, six months, and twelve months after starting biologics. Next, a complete clinical and biological assessment of patients would be made every six months as long as the patient receives treatment. 

The following blood-count-derived inflammatory markers were calculated and further analyzed for all patients: neutrophil-to-lymphocyte ratio (NLR), platelet-to-lymphocyte ratio (PLR), platelet-to-monocyte ratio (PMR), monocyte-to-lymphocyte ratio (MLR), derived neutrophil-to-lymphocyte ration (d-NLR), systemic inflammation response index (SIRI), systemic immune index (SII), and aggregate index of systemic inflammation (AISI). The equations used are depicted in Table 1.

### 2.3. Treatment Response Definition

The following treatment target goals were established: PASI 75 at the three-month hallmark, PASI 90 at the six-month follow-up visit, and PASI 100 at the 12-month visit. Patients achieving PASI 100 at the six-month visit were considered super-responders. Based on whether treatment target goals were met or not, patients were afterward divided into study subgroups and further analyzed. 

### 2.4. Study Outcomes

This paper primarily aims to establish whether blood-count-derived inflammatory markers may serve as predictors of treatment response to biologics. Second, we aimed to identify independent prognostic factors for treatment response and to contour the bio-humoral profile of patients with a proper and sustained favorable response to biologics. To the best of our knowledge, this is the first study to evaluate the usefulness of AISI and d-NLR as blood-count-derived inflammatory markers impacted by biologics and small-molecule inhibitors in psoriasis.

### 2.5. Statistical Analysis

The MedCalc Statistic software for Windows, version 22.023, was used for the statistical analysis. After assessing normality using the Shapiro–Wilk test, categorical variables are expressed as absolute values and percentages. In contrast, continuous variables are depicted using the median or mean values with standard derivations (SD). The Mann–Whitney test for continuous variables or, if referring to categorical variables, the chi-square test was used to evaluate differences between study groups. One-way ANOVA was used to compare data between study groups if the normal distribution was met, and the Kruskal–Wallis test was used for non-normally distributed data. Spearman’s rank correlation coefficient was used to evaluate correlations, while multiple logistic regression was run to identify independent predictors of response to biologics. A *p*-value less than 0.05 was considered statistically significant. 

## 3. Results

### 3.1. Patients’ Clinical Profile

A total of 240 out of 289 eligible patients fulfilled all inclusion criteria and were enrolled in this study. The study group included mainly males (n = 143) and had a mean age of 50.76 ± 14.35 years. Male patients had significantly higher BMI than females (*p* = 0.04), but no differences were noted regarding age (*p* = 0.68). Forty-five patients were diagnosed with psoriatic arthritis, while 103 presented with cardiovascular diseases, such as hypertension or chronic ischemic cardiomyopathy. The highest number of patients were on ixekizumab (n = 61), followed by risankizumab (n = 44) and secukinumab (n = 41). Sociodemographics and treatment of choice are highlighted in Table 2. 

No statistically significant differences were noted between the four visits (initiation, three-month, six-month, and twelve-month hallmarks) regarding platelet (*p* = 0.445), neutrophil (*p* = 0.120), and lymphocyte (*p* = 0.414) count. Regarding the other analyzed parameters, their values varied in dynamics significantly from visit to visit, as seen in Table 3. Activity scores, PASI, PSSI, and NAPSI varied significantly between visits (*p* < 0.001). 

### 3.2. Serological Markers Variation and Disease Severity Evolution

Next, we analyzed whether there is any association (Table 4) between disease severity during the analyzed period of time and the aforementioned blood-count-derived inflammatory markers. As the duration of treatment increased, PLR, NLR, d-NLR, SII, and SIRI decreased. Moreover, as the PASI score decreased, so too did the values of WBC (*p* = 0.006), PLR (*p* = 0.013), d-NLR (*p* = 0.008), and ESR (*p* = 0.045). PSSI values correlated significantly positively with AISI (*p* = 0.038) and SIRI (*p* = 0.028). No correlation was noted between the NAPSI score and the analyzed biomarkers.

### 3.3. Blood-Count-Derived Inflammatory Markers’ Variation Based on the Type of Biologic Used 

For patients treated with TNF-α inhibitors, significant variations in blood-count-derived inflammatory markers were noted between the four analyzed periods of time in terms of d-NLR (adalimumab; *p* = 0.014) and AISI (certolizumab; *p* = 0.04). If referring to IL-17 inhibitors, notable differences were observed for NLR (both ixekizumab and secukinumab; *p* < 0.05), SIRI (ixekizumab; *p*-0.008), MLR (secukinumab; *p* = 0.008) and AISI (secukinumab; *p* = 0.008). For the anti-IL-23 agents, risankizumab led to a significant variation in NLR (*p* = 0.04) during the course of treatment, while guselkumab impacted both NLR (*p* = 0.02) and SII (*p* = 0.04). A comprehensive overview of the analyzed parameters variation during the course of treatment with the analyzed agents is depicted in Table 5.

### 3.4. Clinical and Biological Profile of Responders vs. Nonresponders in Dependence of the Biologic Used 

**Figure 1:**
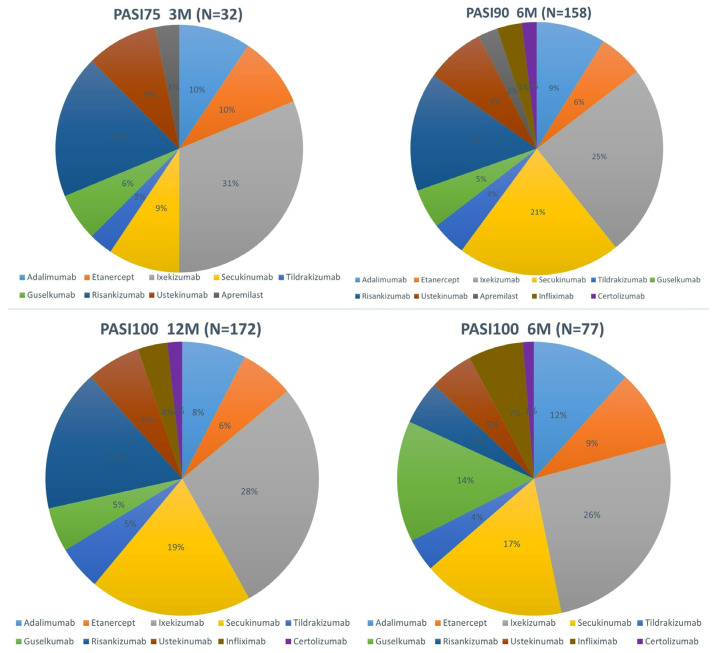
Percentage of patients reaching target goals based on the treatment of choice.

Thirteen percent (n = 32) of patients met the target goal, e.g., PASI75, at the three-month evaluation, while a marked increase (65.83%; n = 158 and 71.66%; n = 172) was noted at the six-month and twelve-month hallmark (as seen in Figure 1). Ixekizumab was the most commonly used treatment for patients achieving treatment target goals at the three-month, six-month, and twelve-month thresholds and was also associated with the highest number of patients being super-responders. 

As depicted in Table 6, blood-count-derived inflammatory markers values did not differ significantly between responders and nonresponders at the three-month and twelve-month hallmark. However, the analyzed markers in both evaluations had lower values in the responders subgroup than in nonresponders. At the 6-month evaluation, for patients meeting the PASI90 target goal, significantly lower values (*p* < 0.05) were noted for all analyzed markers except NLR and PLR.

### 3.5. Blood-Count-Derived Inflammatory Markers Usefulness in Predicting Super-Responder Status

In a multiple logistic regression model (Table 7), higher baseline values of d-NLR and SIRI and scalp involvement (*p* < 0.05) proved to be predictive factors of the super-responder status. However, since the 95% CI for PASI, d-NLR, and SIRI cross over 1, these data should be cautiously interpreted.

## 4. Discussion

Blood-count-derived inflammatory markers have been proven useful in estimating and predicting disease severity in psoriasis [16]. Moreover, existing data highlight their usefulness in predicting psoriasis comorbidities [19] and, thus, guiding the clinician in the treatment choice. With respect to biologics, they have been assessed so far in rheumatic disorders, such as ankylosing spondylitis and psoriatic arthritis [20], rheumatoid arthritis [21], and skin diseases like atopic dermatitis [12] and hidradenitis suppurativa [22]. Biologics’ effects on disease progression in psoriasis have been evaluated in a number of studies [23,24,25] but with conflicting results. Until now, no paper has assessed the usefulness of AISI and d-NLR in predicting responses to biologics. 

Numerous effective biologics targeting various pathways in psoriasis pathogenesis are currently available. Patients with psoriasis present with a high clinical variation during the course of the disease, which could be translated into high variations in the immunological profile of these patients. As such, different patterns in cytokines expression might be noted from patient to patient and afterward impact how a patient reacts to a specific type of treatment. However, cytokines determination is not currently widely and routinely available, and the need for simple, cost-reliable markers to assess the inflammatory status and treatment response is very high. A personalized approach taking into account predictive biomarkers may aid in obtaining a faster and more complete resolution of skin lesions while improving associated comorbidities. 

The highest number of patients included in our study underwent treatment with interleukin inhibitors, specifically IL-17 and IL-23 inhibitors, such as ixekizumab (n = 61), risankizumab (n = 44), and secukinumab (n = 41). In addition to being in the majority, male patients had higher BMI scores than female patients (*p* = 0.04). 

Regarding absolute cell count, WBC and monocyte values decreased linearly and significantly between the four visits (*p* = 0.012); other cell populations did not differ significantly during the course of treatment. 

All blood-count-derived inflammatory markers varied significantly between the four visits (*p* < 0.001); however, only PLR, NLR, d-NLR, PMR, SII, and SIRI presented with a gradual decrease during the treatment period. On the other hand, the PASI score significantly and positively correlated with PLR (rho = 0.175), d-NLR (rho = 0.234), and SII (rho = 0.178), while PSSI correlated with AISI (rho = 0.198) and SIRI (rho = 0.209). NLR is the marker most extensively studied in relation to psoriasis; it was proved to be higher compared to controls [26] and decreased during the course of treatment with biologics [27,28], as seen in our study as well. In a study by Andersen et al. [25], lower baseline values of NLR between responders and nonresponders to anti-TNF-α agents were noted. 

For patients treated with adalimumab, d-NLR significantly varied between the four visits, while in the certolizumab subgroup, the AISI score significantly decreased. In a study published by Albayrak [23], anti-TNF-α agents, such as adalimumab, etanercept, and infliximab, significantly decreased NLR, NMR, and PLR as soon as three months after the start of treatment. Additionally, in the aforementioned study, NLR was correlated with PASI and CRP values, highlighting once more the reliability of this marker in properly assessing the inflammatory status in psoriasis. NLR varied significantly in our study group in patients treated with IL-17 and IL-23 inhibitors. Out of all the parameters that were analyzed, NLR varied significantly the most (in three out of eleven biologics) in our study group. Moreover, high PLR combined with increased serum values of IL-6 and nail involvement was recently proven to be a subclinical indicator of psoriatic arthritis [29]. 

Apremilast did not show any significant variation in the analyzed parameters in the aforementioned period of time, indicating that small-molecule inhibitors might not significantly modify the inflammatory status. However, it should be kept in mind that our study included only one type of small-molecule inhibitor, namely, apremilast, with a limited number of patients (n = 4), and the results for this choice of treatment should be interpreted cautiously. To the best of our knowledge, no study thus far has analyzed the relationship between apremilast and blood-count-derived inflammatory markers in psoriasis; future ideas might include expanding the spectrum of small-molecule inhibitors to include deucravacitinib as well, and to assess their impact in altering blood-count-derived inflammatory markers in psoriasis. 

More than half of patients (65.83%) met their target goals at the 6-month hallmark, while at the 12-month evaluation, 71.66% of them obtained complete clearance of their lesions. Additionally, 32.08% of patients were super-responders, having obtained PASI100 at the 6-month hallmark. Most patients who met target goals underwent treatment with ixekizumab (10, 20, and 48, respectively). Risankizumab and secukinumab closely followed. However, as seen in Figure 1, not only did patients on ixekizumab obtain the fastest response, but the number of patients meeting target goals gradually increased during the follow-up period of time. These data are in accordance with those recently published by Gooderham et al. [30], who identified that patients undergoing treatment with ixekizumab obtained faster skin lesion clearance compared to an IL-23 inhibitor, namely, guselkumab. Moreover, it seems that skin clearance in the head region is achieved faster compared to the trunk, upper, and lower extremities [31,32]. On the other hand, even though risankizumab and secukinumab presented with a slower action in the initial treatment phases, once the target goals were met, they were properly maintained during the follow-up period. Regarding super-responders, the highest number of patients in this group were under treatment with ixekizumab, with slightly fewer patients undergoing treatment with risankizumab and secukinumab. These data suggest that anti-IL-17 and anti-IL-23 agents not only lead to a faster significant clearance of lesions compared to the other classes but have a sustained positive effect, at least, if not more, during a short follow-up period. 

No blood-count-derived inflammatory marker varied significantly between patients who achieved clinical response at the three-month hallmark and those who did not. However, it should be kept in mind that at the three-month evaluation, only 32 patients (13.33%) achieved the treatment goal (as defined, PASI 75), while if referring to the treatment target met at 6 months, 65.83% of patients (n = 158) met clinical resolution, e.g., PASI 90. This matter is also reflected by a gradual, significant decrease in inflammatory markers, such as PLR (*p* = 0.02), MLR (*p* = 0.001), SII (*p* = 0.002), SIRI (*p* = 0.001), and AISI (*p* < 0.001), between responders and nonresponders. 

At the 12-month hallmark, no significant differences between responders and nonresponders were noted between inflammatory markers values. These findings indicate that there is a difference between clinical response and biological response in patients with psoriasis. Treatment should be guided to limit and contain cutaneous lesions and address the possibly associated comorbidities. In this matter, it is once more emphasized the need for a multidisciplinary, comprehensive approach to patients with psoriasis to prevent disease extension, utilizing a careful follow-up of patients during biologics treatment. 

Many countries, including ours, mandate a careful follow-up every 6 months for patients undergoing biologics treatment to limit possible adverse reactions. On the other hand, it must be noted that even for patients considered to be nonresponders at the 12-month hallmark (e.g., patients who did not achieve PASI100), all inflammatory markers values were lower compared to baseline values, indicating that, indeed, biologics decrease the overall inflammatory status of psoriatic patients. Future agreements might shed some light onto what should be considered acceptable regarding treatment targets: Should only PASI100 be desired? Or might PASI90 and even PASI75 be reasonable target goals for both prescribing doctors and patients? 

A total of 77 patients (32.08%) were super-responders, as defined in Section 2, reaching PASI100 at the 6-month hallmark. Most of them were under treatment with ixekizumab (n = 20). Disease severity quantified by the PASI score (OR = 1.03), scalp involvement (OR = 0.29), advanced age (OR = 0.97), higher baseline values of d-NLR (OR = 0.75), and SIRI (OR = 0.20) predicted super-response to treatment. These data should be interpreted cautiously since for three parameters (PASI, d-NLR, and SIRI), the 95% CI crosses over 1, therefore not providing enough evidence. This situation might also be due to the fact that there were a limited number of super-responders (n = 77). Furthermore, it would be incorrect to interpret a positive odds ratio (OR) with a significant *p*-value but with a 95% confidence interval (CI) encompassing the null value as evidence of no association between the exposure and outcome [33]; nevertheless, situations like these should be approached with caution. Early response is also useful for the long-term evolution of such patients. Reaching a PASI of 2 or less within the first six months of starting biologics is associated with a reduced risk of flares and a more stable disease course [34]. 

This is the first paper to assess the usefulness of d-NLR and AISI in predicting treatment response to biologics; our study identified that higher baseline values of d-NLR significantly predicted one patient’s status as a super-responder. On the other hand, AISI significantly decreased during the course of treatment with anti-TNF-α and anti-IL-17 agents, while its values varied between responders and nonresponders at the six-month hallmark. 

This study‘s main limitation is its retrospective design. Future ideas might include prospective patient enrolment. As such, disease severity assessment will be associated with lower bias in data collection. Moreover, our study focused solely on chronic plaque psoriasis; it would be interesting to assess various classical immunosuppressive agents’ effects on blood-count-derived inflammatory markers in pustular or erythrodermic psoriasis. A longer follow-up period would help gain additional data regarding psoriasis evolution under biologics, interleukin levels, and neutralizing antibodies determination. 

## 5. Conclusions

In our study group, all blood-count-derived inflammatory markers varied significantly from visit to visit in evaluating response to biologics. Furthermore, PLR, d-NLR, and SII decreased parallelly with the PASI score in our study group. Most super-responder patients underwent treatment with ixekizumab. If referring to d-NLR, a blood-count-derived inflammatory marker tested for the first time in the literature regarding the treatment of psoriasis with biologics in our paper, it proved to be an integrative part of reaching the super-responder status.

Further, larger-scale studies are necessary to better represent the study population. These cost-effective and simple-to-determine markers can be used to assess systemic inflammation in psoriasis, monitor disease course, and predict patients’ response to biologics. 

## Figures and Tables

**Table 1:** Biomarkers formulas.

Marker	Formula
NLR	Neutrophil count/lymphocyte count [×10^3^/μL]
PLR	Platelet count/lymphocyte count [×10^3^/μL]
PMR	Platelet count/monocyte count [×10^3^/μL]
MLR	Monocyte count/lymphocyte count [×10^3^/μL]
d-NLR	Neutrophil count/(WBC-neutrophil count) [×10^3^/μL]
SIRI	(Neutrophil count × monocyte count)/lymphocyte count [×10^3^/μL]
SII	(Neutrophil count × platelet count)/lymphocyte count [×10^3^/μL]
AISI	(Neutrophil count × monocyte count x platelet count)/lymphocyte count [×10^3^/μL]

NLR, neutrophil-to-lymphocyte ratio; PLR, platelet-to-lymphocyte ratio; PMR, platelet-to-monocyte ratio; MLR, monocyte-to-lymphocyte ratio; d-NLR, derived neutrophil-to-lymphocyte ratio; SIRI, systemic inflammation response index; SII, systemic immune inflammation index; AISI, aggregate index of systemic inflammation.

**Table 2:** Demographic characteristics and treatment of choice of the patients.

Parameter		*p*-Value
**Age (n = 240)**	50.76 ± 14.35	
Male	51.30 ± 13.96	0.68
Female	49.96 ± 14.93	
Gender		
Male	143	
Female	97	
**BMI (n = 240)**	28.89 ± 18.86	
Male	29.23 ± 17.79	0.04
Female	28.40 ± 20.43	
**Psoriatic arthritis (n = 240)**	44	
Male	31	
Female	13	
**Treatment of choice**		
Adalimumab	22	
Etanercept	18	
Infliximab	6	
Certolizumab	6	
Ixekizumab	61	
Secukinumab	41	
Tildrakizumab	11	
Risankizumab	44	
Guselkumab	10	
Ustekinumab	17	
Apremilast	4	

BMI, body mass index.

**Table 3:** Laboratory characteristics of the study population across visits.

Variables	Initial Visit	3-Month Follow-Up	6-Month Follow-Up	12-Month Follow-Up	*p*-Value
WBC	7.12 [6.82–7.32]	6.87 [6.66–7.04]	6.81 [6.47–7]	6.65 [6.48–6.91]	0.012
Platelets	247.35 [235.66–256.33]	242.55 [234–253.66]	242 [232.33–253.33]	247 [235.33–251.67]	0.445
Neutrophils	4.32 [4.12–4.59]	4.10 [3.95–4.31]	3.88 [3.70–4.13]	3.90 [3.72–4.09-	0.120
Lymphocytes	2.02 [1.90–2.12]	2 [1.93–2.08]	2.09 [1.99–2.20]	2.12 [2.10–2.25]	0.414
Monocytes	0.48 [0.45–0.50]	0.50 [0.47–0.52]	0.48 [0.44–0.51]	0.50 [0.48–0.53]	0.016
PLR	122.54 [115.81–127.92]	120.28 [112.86–125.72]	117.72 [109.38–123.36]	116 [108.81–121.73]	<0.001
NLR	2.15 [2.01–2.26]	2.05 [1.84–2.12]	1.82 [1.73–1.95]	1.82 [1.70–1.93]	<0.001
d-NLR	1.57 [1.47–1.65]	1.42 [1.32–1.48]	1.39 [1.29–1.47]	1.38 [1.31–1.48]	<0.001
MLR	0.24 [0.23–0.26]	0.25 [0.23–0.27]	0.23 [0.22–0.25]	0.24 [0.23–0.25]	<0.001
PMR	526.32 [491.32–558.69]	490 [474.01–518.55]	496.72 [466.67–529.65]	483.77 [450–533.69]	<0.001
SII	549.70 [509.36–591.07]	488.51 [457.35–524.22]	450.56 [418.62–475.69]	447.26 [410.83–491.39]	<0.001
SIRI	1.02 [0.94–1.10]	0.99 [0.92–1.09]	0.90 [0.84–0.98]	0.90 [0.85–0.98]	<0.001
AISI	254.76 [227.20–278.32]	242.48 [214.09–279.76]	214.68 [194.11–238.79]	241.48 [212.09–277.76]	<0.001
ESR	12 [10–13]	9 [8–10]	9 [8–10.13]	10 [8–11]	<0.001
Nail involvement (n)	20	14	14	12	0.493
Scalp involvement (n)	42	15	15	15	<0.001
Palmoplantar involvement (n)	18	12	12	10	0.428
PASI	20 [19–20.5]	1.20 [1–1.8]	6.6 [5.5–7.5]	1.56 [1.04–2.09]	<0.001
PSSI	7.12 [4.78–9.47]	0.48 [0.19–0.78]	0.91 [0.32–1.49]	0.09 [0.015–0.17]	<0.001
NAPSI	4.97 [2.23–7.70]	1.49 [0.38–2.61]	1.08 [0.34–1.84]	0.45 [0.13–0.77]	<0.001

NLR, neutrophil-to-lymphocyte ratio; PLR, platelet-to-lymphocyte ratio; PMR, platelet-to-monocyte ratio; MLR, monocyte-to-lymphocyte ratio; d-NLR, derived neutrophil-to-lymphocyte ratio; SIRI, systemic inflammation response index; SII, systemic immune inflammation index; AISI, aggregate index of systemic inflammation; ESR, erythrocyte sedimentation rate; PASI, psoriasis area severity index; PSSI, psoriasis scalp severity index; NAPSI, nail psoriasis severity index.

**Table 4:** Correlation between serological markers and disease severity.

PASI			PSSI		
Marker	rho	*p*-Value	Marker	rho	*p*-Value
WBC	0.175	0.006	AISI	0.198	0.038
PLR	0.159	0.013	SIRI	0.209	0.028
D-NLR	0.234	0.034			
SII	0.178	0.008			
ESR	0.130	0.045	-		

PLR, platelet-to-lymphocyte ratio; D-NLR, derived neutrophil-to-lymphocyte ratio; SIRI, systemic inflammation response index; SII, systemic immune inflammation index; AISI, aggregate index of systemic inflammation; ESR, erythrocyte sedimentation rate; PASI, psoriasis area severity index; PSSI, psoriasis scalp severity index.

**Table 5:** Comparison of blood-count-derived inflammatory markers during treatment course based on the treatment choice.

Variables *	Initial Visit	3-Month Follow-Up	6-Month Follow-Up	12-Month Follow-Up	*p*-Value
**Adalimumab**					
PLR	123.01 [92.79–138.75]	106.84 [83.49–128.04]	98.34 [77.34–116.34]	92.26 [75.92–108.42]	0.98
NLR	2.20 [1.85–2.38]	1.58 [1.34–2.21]	1.42 [1.20–1.65]	1.35 [1.17–1.86]	0.428
d-NLR	1.49 [1.27–1.65]	1.12 [0.92–1.28]	1.11 [0.94–1.29]	1.08 [0.94–1.22]	0.014
MLR	0.24 [0.21–0.29]	0.21 [0.17–0.33]	0.22 [0.18–0.35]	0.24 [0.18–0.29]	0.054
PMR	520.89 [357/98–585.73]	452.80 [274.17–616.10]	448.23 [410.10–484.45]	400.79 [295.81–586.02]	0.060
SII	314.42 [287.26–435.93]	371.42 [301.99–458.99]	372.45 [303.56–456.72]	314.23 [287.26–425.92]	0.283
SIRI	1.15 [0.86–1.45]	0.81 [0.46–1.21]	0.81 [0.47–1.22]	0.79 [0.56–1.21]	0.149
AISI	276.50 [181.43–401.89]	167.91 [112.27–294.04]	178.23 [145.67–234.56]	216.95 [118.34–287.56]	0.15
**Etanercept**					
PLR	142.41 [95.89–182.82]	105.67 [93.85–155.81]	115.11 [94.84–147.41]	124.48 [96.84–156.16]	0.21
NLR	2.21 [1.63–3.21]	1.91 [1.46–2.23]	1.56 [1.26–2.29]	1.54 [1.18–2.06]	0.14
d-NLR	1.65 [1.38–2.29]	1.35 [0.99–1.66]	1.23 [0.99–1.64]	1.16 [0.93–1.64]	0.03
MLR	0.25 [0.22–0.33]	0.25 [0.21–0.35]	0.23 [0.17–0.27]	0.23 [0.17–0.27]	0.25
PMR	555.77 [396.00–728.78]	500.51 [425.16–657.06]	559.25 [388.18–662.07]	569.28 [434.54–693.75]	0.71
SII	574.33 [332.55–886.15]	439.47 [306.33–604.07]	415.99 [324.85–500.69]	389.38 [259.09–559.77]	0.04
SIRI	1.03 [0.72–1.43]	0.86 [0.65–0.99]	0.80 [0.54–0.97]	0.74 [0.50–0.89]	0.01
AISI	247.70 [112.15–422.90]	220.65 [147.27–378.04]	191.89 [131.84–237.09]	177.63 [121.47–232.86]	0.01
**Infliximab**					
PLR	143.59 [98.55–186.66]	117.95 [104.16–239.14]	107.60 [84.56–143.34]	109.26 [96.61–170.79]	0.41
NLR	1.27 [1.58–4.35]	1.81 [1.46–2.91]	1.71 [0.78–2.48]	1.88 [1.38–2.20]	0.45
d-NLR	1.69 [1.16–3.41]	1.45 [1.08–2.17]	1.32 [0.61–2.03]	1.37 [1.02–1.69]	0.32
MLR	0.24 [0.15–0.32]	0.28 [0.19–0.36]	0.22 [0.18–0.30]	0.28 [0.25–0.30]	0.22
PMR	580.44 [372.94–1027.58]	452.30 [387.32–679.04]	503.87 [328.28–671.34]	397.04 [356.02–561.60]	0.12
SII	629.295 [372.53–1192.20]	507.645 [294.46–695.64]	1.01 [0.43–1.11]	432.81 [358.78–614.63]	0.20
SIRI	1.08 [0.70–1.58]	0.785 [0.64–1.70]	1.01 [0.431–1.11]	1.070 [0.87–1.31]	0.17
AISI	254.23 [175.91–470.88]	234.15 [118.12–407.06]	241.12 [133.90–287.77]	285.84 [170.91–381.21]	0.09
**Certolizumab**					
PLR	99.52 [65.05–115.51]	88.07 [54.37–96.40]	97.07 [56.71–123.86]	84.02 [58.47–117.45]	0.48
NLR	2.04 [1.587–2.454]	1.67 [0.94–1.95]	1.86 [1.03–3.54]	1.32 [1.02–2.06]	0.33
d-NLR	1.68 [1.11–1.79]	1.36 [0.75–1.53]	1.38 [0.81–2.15]	1.05 [0.80–1.59]	0.88
MLR	0.23 [0.16–0.30]	0.17 [0.132–0.30]	0.22 [0.09–0.51]	0.22 [0.09–0.26]	0.23
PMR	412.01 [309.10–551.90]	461.54 [194.84–715.28]	532.50 [168.72–746.38]	418.58 [237.50–1004.02]	0.24
SII	420.83 [309.93–592.62]	310.84 [140.07–489.79]	456.93 [216.59–623.88]	341.13 [155.63–496.81]	0.07
SIRI	1.09 [0.79–1.26]	1.69 [0.41–1.31]	0.82 [0.48–3.38]	0.78 [0.37–1.22]	0.07
AISI	204.39 [178.58–299.58]	130.88 [68.59–250.96]	192.91 [102.45–540.86]	154.68 [81.20–356.18]	0.04
**Ixekizumab**					
PLR	124 [108.38–147.07]	121.435 [112.04–140.54]	118.15 [101.70–132.99]	119.09 [107.42–141.70]	0.03
NLR	2.14 [1.93–2.38]	1.89 [1.77–2.28]	1.97 [1.68–2.27]	1.93 [1.84–2.43]	0.04
d-NLR	1.66 [1.47–1.81]	1.57 [1.38–1.67]	1.62 [1.31–1.73]	1.62 [1.41–1.73]	0.21
MLR	0.25 [0.21–0.28]	0.25 [0.22–0.28]	0.24 [0.21–0.27]	0.25 [0.22–0.27]	0.94
PMR	522.5 [470.22–607.38]	506.67 [468.91–576.44]	496.72 [448.60–542.76]	503.51 [451.73–597.79]	0.30
SII	559.89 [507.75–638.92]	508.71 [441.64–577.01]	476 [401.05–525.58]	512.47 [444.83–581.48]	0.29
SIRI	1.08 [0.86–1.27]	0.95 [0.82–1.04]	0.89 [0.74–1.08]	0.98 [0.86–1.25]	0.29
SIRI	1.02 [0.86–1.13]	1.05 [0.88–1.23]	0.99 [0.82–1.22]	0.97 [0.74–1.12]	0.008
AISI	278.32 [213.44–366.69]	216.54 [191.36–284.76]	226.94 [155.31–250.01]	228.16 [194.74–306.96]	0.84
**Secukinumab**					
PLR	125.29 [113.65–151.76]	126.15 [112.78–140.11]	126.47 [110.40–131.59]	119 [108.18–141.65]	0.85
NLR	2.23 [1.88–2.44]	2.07 [1.66–2.56]	1.83 [1.54–2.14]	1.81 [1.42–2.10]	0.005
d-NLR	1.42 [1.36–1.81]	1.475 [1.15–1.80]	1.41 [1.25–1.60]	1.35 [1.11–1.57]	0.154
MLR	0.26 [0.21–0.28]	0.27 [0.23–0.30]	0.25 [0.21–0.29]	0.25 [0.21–0.27]	0.008
PMR	543.26 [467.35–677.31]	481 [412.59–554.94]	461.9 [411.01–493.99]	460 [416.10–573.17]	0.23
SII	520.03 [416.10–645.76]	501.26 [444.11–580.68]	439.78 [403.15–524.57]	412.66 [370.70–507.89]	0.058
SIRI	1.08 [0.86–1.27]	0.95 [0.82–1.04]	0.89 [0.74–1.08]	0.98 [0.86–1.25]	0.29
AISI	241.12 [206.06–277.64]	268.665 [213.56–324.45]	238.55 [186.30–274.01]	217 [187.28–257.58]	0.008
**Tildrakizumab**					
PLR	121.68 [103.15–186.73]	135.79 [98.88–157.40]	124.35 [82.90–157.45]	125.52 [112.18–154.51]	0.23
NLR	2.24 [1.70–3.18]	2.3 [1.54–3.46]	2.42 [1.36–2.86]	2.18 [1.57–2.95]	0.86
d-NLR	1.42 [1.23–2.35]	1.63 [1.16–2.41]	1.69 [1.01–2.13]	1.57 [1.25–2.06]	0.78
MLR	0.27 [0.19–0.33]	0.28 [0.20–0.31]	0.25 [0.17–0.32]	0.24 [0.17–0.38]	0.71
PMR	474 [440.68–720.67]	485 [438.03–579.58]	530.43 [354.84–631.98]	520 [363.72–785.12]	0.32
SII	611.29 [371.10–709.35]	574.39 [439.87–792.95]	554.68 [284.14–632.80]	508.38 [451.50–688.02]	0.72
SIRI	0.85 [0.67–1.51]	1.19 [0.83–1.57]	1 [0.67–1.47]	0.91 [0.69–1.61]	0.88
AISI	266.63 [158.77–363.59]	271.6 [163.98–412.84]	206.97 [153.79–393.27]	205.03 [160.54–341.37]	0.99
**Risankizumab**					
PLR	113.17 [103.35–124.92]	120.62 [101.75–133.97]	111.915 [99.65–19.96]	115.17 [102.28–124.96]	0.16
NLR	1.9 [1.451–2.23]	2.185 [1.87–2.42]	1.735 [1.53–1.89]	1.75 [1.43–1.99]	0.04
d-NLR	1.405 [1.14–1.71]	1.625 [1.43–1.77]	1.295 [1.14–1.47]	1.26 [1.05–1.51]	0.54
MLR	0.225 [0.18–0.25]	0.25 [0.21–0.28]	0.21 [0.18–0.24]	0.22 [0.19–0.25]	0.81
PMR	545 [491.56–657.59]	482.8 [427.38–621.04]	530.78 [459.55–635.14]	511.76 [425.13–670.27]	0.62
SII	464.905 [380.01–556.99]	501.505 [444.36–605.69]	415.23 [345.09–510.48]	435.86 [367.68–544.54]	0.33
SIRI	0.935 [0.670–1.01]	1.145 [0.891–1.23]	0.79 [0.610–0.98]	0.88 [0.690–1.02]	0.20
AISI	214.425 [153.78–290.81]	288.22 [211.08–322.75]	188.23 [155.43–248.86]	219.33 [159.79–267.39]	0.15
**Guselkumab**					
PLR	120.225 [100.15–189.98]	136.48 [92.11–61.93]	120.615 [80.71–159.89]	115.155 [80.98–82.98]	0.80
NLR	2.18 [1.75–4.20]	2.11 [1.52–2.80]	1.75 [1.46–2.09]	1.95 [1.64–2.28]	0.02
d-NLR	1.53 [1.31–2.12]	1.61 [1.12–1.82]	1.35 [0.96–1.57]	1.4 [1.01–1.55]	0.45
MLR	0.26 [0.21–0.39]	0.24 [0.18–0.26]	0.25 [0.10–0.27]	0.24 [0.18–0.27]	0.12
PMR	468.16 [349.90–828.32]	551.25 [381.29–832.06]	589 [356.09–846.16]	533.61 [401.86–719.85]	0.53
SII	717.79 [510.73–1026.12]	534.54 [390.93–1037.29]	464.85 [373.21–591.27]	551.27 [349.65–848.11]	0.04
SIRI	1.26 [0.88–2.80]	1.06 [0.74–1.47]	0.85 [0.45–1.21]	1.02 [0.75–1.46]	0.07
AISI	393.36 [274.36–715.36]	291.51 [184.81–470.05]	251.93 [134.54–295.49]	276.81 [175.55–499.80]	0.09
**Ustekinumab**					
PLR	155.11 [104.69–176.66]	122.69 [98.141–63.520]	127.41 [98.76–51.63]	107.69 [83.93–49.17]	0.08
NLR	3.03 [1.47–3.61]	2.4 [1.84–2.87]	1.95 [1.51–2.73]	1.93 [1.29–2.19]	0.08
d-NLR	2.19 [1.52–2.38]	1.82 [1.43–2.11]	1.72 [1.19–2.04]	1.6 [1.24–1.86]	0.50
MLR	0.27 [0.22–0.43]	0.25 [0.18–0.33]	0.23 [0.15–0.33]	0.23 [0.14–0.30]	0.98
PMR	512.5 [366.42–763.31]	490 [415.03–675.81]	662.96 [335.65–732.27]	543.48 [416.98–620.79]	0.43
SII	668.51 [452.76–997.77]	524.38 [407.29–708.24]	530.26 [393.45–695.04]	440.12 [356.07–572.94]	0.38
SIRI	1.15 [0.85–1.94]	1 [0.60–1.94]	1.05 [0.51–1.52]	0.78 [0.68–1.56]	0.48
AISI	274.09 [184.63–596.28]	244.26 [154.35–377.77]	217.01 [140.54–383.16]	188.5 [160.67–582.63]	0.66
**Apremilast**					
PLR	137.60 ± 22.64	140.17 ± 29.29	133.44 ± 20.92	129.52 ± 24.67	0.77
NLR	1.77 ± 0.635	1.84 ± 0.57	2.12 ± 0.38	1.93 ± 0.56	0.67
d-NLR	1.31 ± 0.41	1.31 ± 0.23	1.60 ± 0.33	1.36 ± 0.16	0.38
MLR	0.22 ± 0.09	0.27 ± 0.12	0.23 ± 0.09	0.23 ± 0.10	0.91
PMR	684.32 ± 260.47	588.48 ± 243.37	624.27 ± 249.77	654.06 ± 274.05	0.77
SII	443.36 ± 206.54	457.76 ± 206.71	552.72 ± 193.12	513.89 ± 212.43	0.99
SIRI	0.77 ± 0.47	0.96 ± 0.67	0.95 ± 0.37	0.95 ± 0.59	0.43
AISI	194.02 ± 116.23	248.72 ± 201.88	252.48 ± 117.71	262.15 ± 161.23	0.61

PLR, platelet-to-lymphocyte ratio; NLR, neutrophil-to-lymphocyte ratio; d-NLR, derived neutrophil-to-lymphocyte ratio; MLR, monocyte-to-lymphocyte ratio; PMR, platelet-to monocyte ratio; SII, systemic immune inflammation index; SIRI, systemic inflammation response index; AISI, aggregate index of systemic inflammation; * is expressed as mean ± SD for normally distributed data (Apremilast) and as median with 95% CI for non-normally distributed data.

**Table 6:** Comparison of blood-count-derived inflammatory markers between responders and nonresponders.

Variable	PLR	NLR	d-NLR	MLR	PMR	SII	SIRI	AISI
**PASI75 3M**								
**Responder**	108.565 [98.17–120.65]	1.97 [1.73–2.42]	1.34 [1.20–1.55]	0.23 [0.19–0.28]	493.7 [396.60–575.78]	506.205 [426.29–576.00]	1.000 [0.80–1.23]	250.590 [177.03–372.85]
**Nonresponder**	122.66 [113.73–129.31]	2.015 [1.81–2.13]	1.535 [1.43–1.60]	0.25 [0.20–0.27]	491.43 [473.01–525.13]	488.51 [454.53–520.99]	0.985 [0.89–1.08]	241.13 [209.85–283.21]
***p*-value**	0.18	0.64	0.48	0.58	0.82	0.55	0.54	0.72
**PASI90 6M**								
**Responder**	108.24 [98.99–115.65]	1.80 [1.74–2.00]	1.41 [1.29–1.56]	0.25 [0.23–0.26]	480.26 [447.80–518.00]	472.04 [434.86–513.82]	0.98 [0.89–1.07]	158.97 [141.46–191.91]
**Nonresponder**	121.74 [114.58–127.65]	1.85 [1.58–1.97]	1.35 [1.15–1.47]	0.22 [0.18–0.24]	522.05 [477.84–591.78]	404.82 [338.55–450.37]	0.71 [0.63–0.87]	244.35 [227.90–269.58]
***p*-value**	0.02	0.15	0.06	0.001	0.31	0.002	0.001	<0.001
**PASI100 12M**								
**Responder**	115.385 [108.526–122.400]	1.83 [1.700–1.987]	1.375 [1.273–1.480]	0.24 [0.230–0.260]	494.205 [450.000–534.041]	442.445 [398.253–499.176]	0.88 [0.813–0.980]	217.73 [195.332–242.703]
**Nonresponder**	119.145 [102.830–129.133]	1.77 [1.632–1.930]	1.405 [1.232–1.520]	0.23 [0.204–0.260]	478.935 [425.492–593.406]	453.725 [417.552–539.350]	0.965 [0.754–1.026]	227.555 [192.587–264.591]
***p*-value**	0.48	0.98	0.49	0.57	0.74	0.77	0.91	0.94

PLR, platelet-to-lymphocyte ratio; NLR, neutrophil-to-lymphocyte ratio; d-NLR, derived neutrophil-to-lymphocyte ratio; MLR, monocyte-to-lymphocyte ratio; PMR, platelet-to-monocyte ratio; SII, systemic immune inflammation index; SIRI, systemic inflammation response index; AISI, aggregate index of systemic inflammation.

**Table 7:** Predictors of super-responder status.

Parameter	OR	95% CI	*p*-Value
Age	0.97	0.95–0.99	0.01
Scalp involvement	0.29	0.11–0.78	0.02
PASI	1.03	0.99–1.05	0.04
d-NLR	0.75	0.46–1.33	0.05
SIRI	0.20	0.02–2.17	0.04

PASI, psoriasis area severity index; d-NLR, derived neutrophil-to-lymphocyte ratio; SIRI, systemic inflammation response index.

## Data Availability

All data presented can be made available upon request.
